# The Many Potential Fates of Non-Canonical Protein Substrates Subject to NEDDylation

**DOI:** 10.3390/cells10102660

**Published:** 2021-10-05

**Authors:** Kartikeya Vijayasimha, Brian P. Dolan

**Affiliations:** Department of Biomedical Sciences, Carlson College of Veterinary Medicine, Oregon State University, Corvallis, OR 97331, USA; vijayask@oregonstate.edu

**Keywords:** NEDD8, ubiquitin-like proteins, post-translational modifications

## Abstract

Neuronal precursor cell-expressed developmentally down-regulated protein 8 (NEDD8) is a ubiquitin-like protein (UBL) whose canonical function involves binding to, and thus, activating Cullin–Ring finger Ligases (CRLs), one of the largest family of ubiquitin ligases in the eukaryotic cell. However, in recent years, several non-canonical protein substrates of NEDD8 have been identified. Here we attempt to review the recent literature regarding non-canonical NEDDylation of substrates with a particular focus on how the covalent modification of NEDD8 alters the protein substrate. Like much in the study of ubiquitin and UBLs, there are no clear and all-encompassing explanations to satisfy the textbooks. In some instances, NEDD8 modification appears to alter the substrates localization, particularly during times of stress. NEDDylation may also have conflicting impacts upon a protein’s stability: some reports indicate NEDDylation may protect against degradation whereas others show NEDDylation can promote degradation. We also examine how many of the in vitro studies measuring non-canonical NEDDylation were conducted and compare those conditions to those which may occur in vivo, such as cancer progression. It is likely that the conditions used to study non-canonical NEDDylation are similar to some types of cancers, such as glioblastoma, colon and rectal cancers, and lung adenocarcinomas. Although the full outcomes of non-canonical NEDDylation remain unknown, our review of the literature suggests that researchers keep an open mind to the situations where this modification occurs and determine the functional impacts of NEDD8-modification to the specific substrates which they study.

*Ha, ha, ha, thou entanglest thyself in thine own work like a silkworm*

-John Webster, The White Devil

## 1. Introduction

Ubiquitin and ubiquitin-like proteins are small proteins expressed by eukaryotic cells that can be covalently coupled to other proteins within the cell, altering their function. Ubiquitin was the first of these proteins to be discovered. The most often described function of ubiquitin is in protein degradation. The addition of ubiquitin to a target protein in the cell allows for proteasome-mediated degradation. However, the results of ubiquitin conjugation are far more diverse than originally appreciated. The first protein to be identified as a substrate for ubiquitin was a histone [1,2] and it is now recognized that the modification of histone proteins by single ubiquitin monomers is associated not with the degradation of histones, but rather alterations in transcriptional activity [3]. Furthermore, the way ubiquitin is conjugated to a protein, through different lysine residues, or chain structures, can alter the consequences for the substrate [4]. Therefore, ubiquitin conjugation can have many different outcomes beyond protein degradation.

There as many as 20 proteins in eukaryotes which are similar to ubiquitin and termed ubiquitin-like proteins or UBLs [5]. The function of UBL-conjugation is generally less-well understood compared to ubiquitin. The UBLs ATG8 and ATG12 are necessary for the formation of functional autophagosomes [6]. Proteins from the small ubiquitin-like modifier (SUMO) family are known to interact with a variety of substrates, and is necessary for maintaining genome stability and transcription activity [7]. Interferon-stimulatory gene 15 (ISG15) is induced upon viral infection and covalently modifies newly synthesized proteins [8,9]. Given the vastly different outcomes for a substrate that occur following modification with ubiquitin, it is worth keeping an open mind regarding UBL-modification and what the functional consequences may be to the modified substrate.

**Figure 1:**
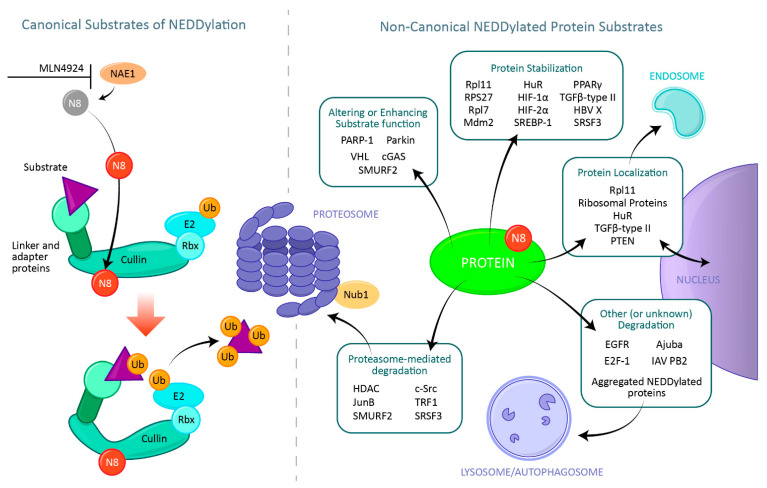
Canonical and non-canonical NEDD8 substrates. Canonical NEDDylation refers to the post-translational modification of the Cullin family of proteins with NEDD8. NEDD8 is first activated by binding to the E1 NEDD8 activating enzyme, a process which can be blocked by the small molecule MLN4924. NEDD8 is then transferred via E2 and E3 enzymes to the Cullin protein, which in turn activates the CRL allowing it to transfer ubiquitin molecules to its designated substrate, leading to the substrate degradation. Non-canonical NEDDylation occurs when NEDD8 is covalently attached to protein substrates other than Cullin molecules. Following NEDDylation these proteins can adopt different fates, such as increased stability, increased enzymatic activity, changes in subcellular location, and degradation. Examples of substrates subject to these outcomes are listed. The list presented here is by no means exhaustive.

Here we discuss potential non-canonical substrates for the UBL neuronal precursor cell-expressed developmentally down-regulated protein 8, also known as NEDD8. The canonical function of NEDD8 is to modify a specific family of proteins, known as Cullins. However, other substrates for NEDD8 have also been identified. We will discuss what substrates have been identified thus far as well as the possible outcomes of non-canonical NEDDylation (Figure 1).

## 2. NEDD8 Modification of Cullins: The Canonical NEDD8 Function

NEDD8, as with ubiquitin and other UBLs, is added to its target substrate through a series of steps. The precursor form of NEDD8 is first modified to expose a C-terminal glycine residue which forms a covalent bond with the E1 NEDD8 activating enzyme, NAE1. NEDD8 is subsequently transferred to an E2 conjugating enzyme and in coordination with an E3 ligase, attached to its substrate. NEDDylation can be reversed by the action of deNEDDylating enzymes such as the COP9 signalosome (CSN) [10] and sentrin-specific protease 8 (SENP8, also known as DEN1 or NEDP1) [11,12]. Of great importance to the field of NEDD8 biology was the discovery of a small molecule, MLN4924, which can selectively inhibit the action of NAE1 and therefore eliminate NEDDylation within a cell [13]. MLN4924 (Pevonedistat) is currently being used in multiple clinical trials for the treatment of a variety of cancers, which has been recently reviewed elsewhere [14,15] and will not be discussed further here.

The first substrate of NEDD8 identified was found in the yeast *Saccharomyces cerevisiae*. NEDD8, also known as Rub1 (related to ubiquitin 1), was found to modify Cdc53 [16,17], the yeast ortholog of the human Cullin protein CUL1. Cullins interact with specific RING proteins to form Cullin–Ring ligases (CRLs). These ligases, with interactions of other proteins, are E3 ubiquitin ligases which can target hundreds of substrates for ubiquitination. The addition of NEDD8 to the Cullin component of the CRL is an important step for the proper function of the E3 ligase [18]. In the absence of Cullin NEDDylation, the substrate binding domains are separated from the ubiquitin conjugating E2 domain. However, upon binding of NEDD8 to the Cullin, the CRL undergoes a conformational change which allows the substrate and E2 to come into close proximity, facilitating the transfer of ubiquitin to the substrate [19,20], though it should also be noted that the NEDDylation may also lead to destabilization and degradation of the Cullin protein [21]. NEDD8 bound to Cullin proteins also prevents the binding of Cullin-associated and NEDDylation-dissociated protein 1 (CAND1) [22], a protein which facilitates the exchange of substrate receptors from the CRL (reviewed in [23]). For a more detailed description of canonical NEDD8-Cullin interactions, the reader is encouraged to consult a compilation of recently published, in-depth review articles [15,23,24,25,26,27,28,29,30,31,32,33,34,35,36,37,38,39,40,41]. The remainder of this article will focus instead on instances where NEDD8 modification has been detected on other proteins and what function this may have in the cell (see Figure 1 for a summary of canonical and non-canonical NEDD8 substrates).

## 3. Proteomic Analysis for NEDD8-Substrate Identification

After the identification of Cullins as a substrate for NEDDylation and the elucidation of the mechanisms underlying how NEDD8-modification of Cullins leads to activation of CRLs, a variety of proteomic approaches were conducted to determine what other substrates of NEDD8 may exist. It has been over a decade since the first proteomic studies were undertaken to identify NEDDylated substrates [42,43,44,45]. These studies identified anywhere from a handful to several hundred different substrates for NEDDylation. Not surprisingly, Cullin proteins as well as enzymes involved in NEDD8 activation and conjugation were identified in these studies. However, several other proteins were identified that were not generally considered to be targets of NEDDylation. In many of these studies, NEDD8-conjugated proteins were isolated by overexpression of an epitope-tagged NEDD8 from DNA vectors transfected into cells. The tagged proteins were then isolated by immunoprecipitation prior to mass spectroscopy analysis to determine their identity. However, alterations to the ratio of NEDD8 to ubiquitin, which would occur if NEDD8 is over-expressed following transfection, can result in the incorporation of NEDD8 into ubiquitin chains, creating the potential for artifacts [46]. Recent studies have sought to overcome this potential problem using a variety of techniques. When levels of epitope-tagged NEDD8 was reduced in cells, afforded using a deconjugation-resistant form of NEDD8, hundreds of protein-targets capable of being modified by the addition of NEDD8 were still identified [47]. In addition, a recent paper was able to identify NEDDylated substrates without the need to overexpress NEDD8 at all. Vogl et al. used a gene knock-in approach to generate a mutant form of NEDD8 that could be subjected to enzymatic digestion, purification, and mass spectroscopy analysis to identify specific NEDDylated substrates [48]. In addition to Cullins, the authors found several hundred other NEDDylated substrates in the HEK cells, and importantly, the levels of substrate NEDDylation could be altered either by treatment with MLN4924 or by deletion of the deNEDDylating enzyme NEDP1. Using a similar approach, Lobato-Gil found hundreds of proteins modified by both typical NEDDylation and under conditions that promote atypical NEDDylation [49]. Taken together, these studies demonstrate that proteins other than Cullins are subject to post-translational modification by NEDD8.

Proteomic studies have been extended beyond the total proteome and used to analyze NEDDylated proteins in subcellular compartments. Jayabalan et al. isolated and identified NEDD8-conjugated proteins from stalled ribosomes associated with stress granules [50] while Maghames et al. identified numerous NEDDylated proteins that associate with nuclear aggregates following heat shock of cells [51]. Li et al. provide recent evidence that the NEDDylation status of 81 proteins can promote localization either to, or away, from the nucleus [52]. Taken together these studies have demonstrated that many different proteins can be modified by the addition of NEDD8. With these data in hand researchers can begin to determine what the consequences of NEDD8 modification may be.

## 4. Ribosomal Protein NEDDylation

Ribosomal proteins were identified as substrates for NEDDylation over a decade ago [43] by Xirodimas et al., though the functional outcome of NEDDylation is not fully understood. A study by Sundqvist et al. first reported that NEDD8 conjugation to 60S ribosomal protein 11 (L11) protected L11 from degradation and this in turn helped to stabilize p53 levels within the cell [53]. The NEDDylation state of L11 influences its ability to interact with the ubiquitin ligase Mdm2 which in turn prevents ubiquitination and degradation of p53 [54]. This process was controlled by Myeov2, which can reverse L11 NEDDylation changing the nuclear localization of L11, allowing L11 to localize outside of the nucleus and regulate p53 activity [55]. MDM2 has also been shown to NEDDylate the ribosomal proteins RPS27and RPS27L which conferred stability to the protein and promoted cancer cell survival [56]. Recent work has provided some significant clues as to how and why ribosomal proteins may be the target of NEDD8 modification, especially during periods of stress to the cell. Maghames et al. used a SILAC-based approach to identify substrates of NEDDylation and found many ribosomal proteins that, upon proteotoxic stress, aggregated in the nucleus [51]. Coupling of NEDD8 to the aggregated proteins likely preserved proteasome function in the nucleus and indeed, Rpl7 was not degraded by the proteasome upon NEDDylation. Overall, these reports suggest that NEDD8-conjugation to ribosomal proteins during times of cellular stress can be beneficial to the cell either by activating the tumor suppressor p53 or by preserving components of protein synthesis until such time as the stress is relieved.

## 5. Protein Degradation

The role of NEDD8 in protein destabilization and degradation is far more murky than other known outcomes of both typical and atypical NEDDylation. Oved et al. were the first to show that NEDD8 conjugation could lead to a protein’s destruction [57], when they demonstrated activated EGFR was NEDDylated and subsequently degraded, likely in lysosomal compartments. In recent years, several additional NEDD8-substrates have been reported to undergo degradation. Pandey et al. demonstrated that HDAC2 can be NEDDylated upon exposure to oxidized LDL and degraded by the proteasome [58] while Lai et al. found HDAC1 expression could be reduced upon NEDD8 conjugation in acute myelogenous leukemia cells [59]. Li et al. found that JunB can be NEDDylated and degraded in a ubiquitin dependent manner [60], similar to results reported by Shu et al. regarding the addition of NEDD8 to the ubiquitin ligase SMURF2 [61] and from Lee et al. who found that c-Src is poly-ubiquitinated and degraded following NEDDylation [62].

The protein NEDD8 Ultimate Buster-1 (NUB1) was identified as a regulator of NEDD8 in cells which allowed for proteasomal degradation of free NEDD8 and NEDD8 conjugated proteins [63,64]. NUB1 is a splice variant of a larger protein termed NUB1L, which has an additional Ubiquitin-Association domain (UBA). NUB1L retains the ability to shuttle NEDD8 and NEDD8-conjugated proteins to the proteasome [65,66], and the UBA domains of NUB1L has been shown to interact with the UBL FAT10 [67,68,69] to facilitate proteasomal degradation of FAT10-conjugates substrates. Tanji et al. were the first to show that NUB1 (through its C-terminal domain) interacts directly with the 19S proteasome subunit Rpn10/S5a [70]. Rani et al. found a similar association between NUB1L and Rpn10/S5a, though an association between NUB1L and Rpn1 was also detected [71]. Interestingly, the UBA domain of NUB1L interacted with Rpn1 but not Rpn10, suggesting that NUB1 and NUB1L may have multiple ways to dock with the proteasome. Liu et al. found that NUB1/NUB1L can also interact with p97, a AAA-ATPase which is involved in numerous cellular functions [65]. Deletion of the p97 interacting domain in NUB1/NUB1L prevented degradation of NEDDylated substrates, suggesting that p97 was involved in proteasome-mediated degradation. Taken together, these studies provide a plausible mechanism to explain how NEDDylated substrates can be degraded by the proteasome.

Several proteins have been identified as targets for NUB1-dependant degradation, and in some cases, degradation can occur independently of substrate NEDDylation. The telomeric protein TRF1 was found to be degraded by the proteasome in a NUB1 dependent process, and while degradation of TRF1 could occur following NEDDylation [72], it was also possible to be degraded following MLN4924 treatment suggesting both NEDD8 dependent and independent pathways of degradation. The degradation of Huntington protein (Htt) requires NUB1, but is thought to occur through an indirect mechanism whereby NUB1 interacts with Htt and recruits a NEDDylated CRL directly to Htt, facilitating poly-ubiquitination and proteasome-mediated degradation of Htt [73]. The NEDD8 interacting domain of NUB1 can also be used to interact with non-NEDDylated proteins to induce degradation, such as α-synphilin, an important mediator of Lewis body-like inclusion formation [74]. The requirement for NUB1 may also be generalized to misfolded proteins subject to atypical NEDDylation. Li et al. demonstrated that degradation of both a model misfolded protein and cardiomyopathy-linked misfolded protein, CryAB(R120G) were dependent on NUB1 expression [75].

Other substrates were found to be degraded upon NEDDylation but independently of proteasome activity. Ajuba was identified as a substrate for NEDD8 in hepatic cell carcinoma cell lines and its degradation was inhibited upon treatment with MLN4924, but not proteasome inhibition [76]. Loftus et al. determined that the transcription factor E2F-1 was NEDDylated in a MLN4924 dependent manner and that NEDDylation decreased levels of E2F-1 in cells, though it is unknown how E2F-1 was degraded [77]. We recently tested if the direct conjugation of NEDD8 to the N-terminus of a protein would lead to degradation. N-terminal fusion proteins were created where the C-terminal glycine residues of NEDD8 were mutated to alanine which prevented removal of NEDD8 from the target protein, such as GFP or chicken ovalbumin. This modification resulted in an increased rate of degradation of the fusion protein demonstrating that N-terminal modification by NEDD8 can lead to a proteins degradation similar to N-terminal fusion of ubiquitin [78]. Interestingly, degradation of these substrates was blocked by both proteasome inhibition and by inhibition of autophagy, suggesting that there are multiple ways for cells to degrade NEDDylated proteins. Indeed Ghosh et al. have identified that the aggregate-prone form of Htt is NEDDylated and interacts with the UBA domain of huntingtin interacting protein K (HYPK). HYPK also interacts with the autophagy factor LC3 and together, induce the degradation of aggregated Huntington protein via the autophagy pathway [79]. Kim et al. have recently shown that under stressful conditions which promote atypical NEDDylation in the cytoplasm, HDAC6 can directly interact with non-canonical targets of NEDDylation and induce aggregate formation [80]. Following HDAC6 interaction with NEDDylated proteins, p62 and LC3 were recruited to promote the formation of aggresome-like bodies, which were ultimately removed through by autophagy. These data demonstrate that non-canonical NEDDylation may lead to non-proteasome dependent degradation of NEDDylated substrates.

Combined, these studies demonstrate that the conjugation of NEDD8 to non-Cullin proteins can lead to a substrate’s degradation. This could occur via the ubiquitin-proteasome system, through lysosomal degradation, or even via the autophagy pathway. Therefore, it is important to consider that multiple different mechanisms exist in the cell for removing NEDD8-coupled proteins.

## 6. Protein Stabilization

In some instances, the covalent modification of a protein with NEDD8 can enhance its stability. Already mentioned were ribosomal proteins, whose stability can be enhanced by NEDDylation and sequestration in nuclear aggregates; however, the stability of several other proteins is enhanced by post-translational modification with NEDD8. The ubiquitin ligase MDM-2 was one of the first non-Cullin proteins to be identified as a substrate for NEDDylation [81] and follow up studies determined that MDM-2 stability can be controlled by NEDDylation [82], which, in turn, can regulate p53 activation. DNA-damaging chemotherapeutic agents could increase levels of NEDP1, which reversed MDM-2 NEDDylation and allowed for destabilization of MDM-2 and activation of the p53 pathway. MDM-2 can also induce the NEDDylation of Hu antigen R (HuR), which helps to stabilize HuR allowing for access to the nucleus [83].

Multiple other proteins have been identified which gain stability following covalent modification with NEDD8. Hypoxia-induced factors (HIF-1α and HIF-2α) were identified as a substrate for NEDD8 modification [84] and HIF-2α levels increased upon NEDDylation. Another study noted that following induction of hypoxia, NEDDylation helped to stabilize levels of HIF-1α in tumor cells, which presumably enhances the ability of tumors to survive in hypoxic conditions [85]. A likely explanation for enhanced protein stability following the addition of NEDD8 to a substrate is that NEDDylation protects substrates from ubiquitination. Recently, Heo et al. demonstrated that sterol regulatory element-binding protein 1 (SREBP-1) stability is regulated by NEDDylation in multiple tumor cell lines [86]. Treatment with either MLN4924 or siRNA depletion of NEDD8, decreased levels of SREBP-1, presumably by allowing ubiquitination of SREBP-1. The increase in SREPB-1 protein levels afforded by NEDD8 conjugation resulted in enhanced proliferation and migration of tumor cells. Similarly, NEDDylation of the electron transport flavoproteins A and B promoted stabilization of both proteins in hepatic cells, which antagonized ubiquitination of the substrates and prevented degradation [87]. Peroxisome proliferator-activated receptor gamma (PPAR*γ*) stability was also dependent on NEDDylation in preadipocytes, and enhancing NEDDylation through NEDD8 overexpression prevented PPAR*γ* ubiquitination [88]. NEDD8 stabilization of proteins extends to cell surface proteins. NEDDylation of TGFβ-type II receptor promoted the trafficking of the receptor to early endosomes, rather than caveolin containing compartments where the receptor would be ubiquitinated and degraded [89]. Taken together these studies demonstrate that post-translational modification of a protein with NEDD8 can stabilize the substrate, perhaps by protecting the targeted protein from ubiquitination.

The apparently conflicting data that NEDD8 can promote both protein degradation and stabilization points to the complexity of cell biology which is often resistant to our desire to classify how systems are supposed to work. Efforts to define rules as to why some modified proteins are degraded and others will be difficult to elucidate. For instance, one may hypothesize that viral proteins (which are expressed in large quantities following infection and foreign to the infecting cell) would be governed by the same rules, but even here we run into resistance. The influenza protein PB2 can be destabilized by NEDD8 conjugation, leading to reduced replication of influenza virus [90]; however, hepatitis B virus-encoded X protein is stabilized upon NEDDylation [91]. The picture can be even more confusing when NEDDylation of the same substrate appears to yield conflicting results. Kumar et al. report that NEDDylated SRSF3 was degraded, a process which can be prevented by treating cells with a proteasome inhibitor [92]. In this study, lysine 11 of SRSF3 was identified as the site of NEDD8 addition and necessary for its degradation. However, Jayabalan et al. identified lysine 85 as the site for di-NEDDylation of SRSF3 [50], and the addition of NEDD8 to this residue was necessary for the formation of stress granules in the cytoplasm of cells. Although the stability of NEDD8-modified SRSF3 was not directly measured in this study, components of stress granules are thought to be relatively stable within cells, at least until the stressor is removed. K11 modification with NEDD8 was enhanced upon treatment of cells with palmitic acid and could be prevented by treating cells with an antioxidant. In contrast, K85 NEDDylation and stress granules formed upon treatment of cells with arsenite, but not other stressors such as clotrimazole and thapsigargin. These studies raise the distinct possibility the NEDD8 modification not only leads to different impacts upon protein stability, but suggests that the types of stress encountered by cells can result in different NEDD8-dependant responses, and that the type and location of NEDD8 modification on a substrate has different outcomes.

## 7. Alterations and Enhancement of a Substrates Function

Several non-canonical NEDD8 substrates have been identified whose stability or degradation are unaltered by NEDDylation, but rather their function is changed or enhanced. Stickle et al. identified von Hippel–Lindau (VHL) as a substrate for NEDDylation [93] which was necessary for promoting assembly of the fibronectin matrix. NEDD8 was shown to be a “molecular switch” whereby NEDDylation of VHL prevents its incorporation into a CRL and promotes its ability to interact with fibronectin [94]. Several reports have also demonstrated that Parkin activity was enhanced following NEDDylation [95,96,97]. Interestingly, VHL, Parkin, and Mdm2 (discussed previously) are all E3 ligases which contain a RING domain; however, NEDD8-enhancement of activity is not limited to RING ligases. The innate immune cytosolic DNA sensor cyclic GMP-AMP (cGAMP) synthetase (cGAS) can be NEDDylated leading to enhanced binding to viral DNA [98]. NEDD8 may also bind to Caspase-1, leading to Caspase-1 activation and blocking NEDDylation with MLN4924 reduced maturation of the inflammatory cytokine IL-1β [99]. These studies provide examples where NEDD8 modification of non-Cullin substrates can enhance their activities or even alter their function.

## 8. Alterations to NEDD8 Levels in Cells under Physiological Conditions

A potential complicating factor in our understanding of NEDD8 substrates arises from the knowledge that NEDD8 can be used by the cell in place of ubiquitin, a process termed atypical NEDDylation. This can occur when NEDD8 levels are increased within the cell, or when ubiquitin levels fall. Interestingly, the E1 activating enzyme for ubiquitin, UBA1, can then activate NEDD8 and allow it to be incorporated into the ubiquitin conjugation pathways [49,51,75,100,101]. When this occurs, mixed ubiquitin and NEDD8 chains can be detected within substrates. This finding is important when considering what constitutes actual NEDD8 substrates, as overexpression of epitope-tagged NEDD8 is often used in proteomic analysis of cells to identify NEDD8 targets. Although we should not dismiss the possibility of artifacts created under these circumstances [46], it is important to consider that these exact conditions may occur in biologically and clinically relevant settings. Recently Zou and Zhang expertly reviewed both canonical and non-canonical roles of NEDD8 in metabolism and immunity. Through the use of promoter analysis software, they determined that components of NEDDylation have the necessary elements in their promoters for up-regulation during periods of cell stress or inflammation [102] suggesting that during certain disease states, NEDD8 levels will be increased, reflecting the conditions used in vitro to identify non-Cullin substrates resulting from atypical NEDDylation.

A primary disease for consideration would be cancer where dysregulated biochemical pathways are commonly detected. Increased NEDD8 levels along with increased levels of enzymes in the NEDD8 activation and conjugation pathway such as NAE1 have been detected in numerous cancer types, such as pancreatic cancer [103], osteosarcoma [104], bladder [105], and colorectal cancers [106] to name but a few. Interestingly, in the case of colorectal cancers, a non-canonical target of NEDD8 was identified [106]. The ubiquitin ligase SMURF1 was auto-NEDDylated, and following NEDDylation the ubiquitin ligase activity of SMURF1 was enhanced, similar to other E3 ligases mentioned above. Increasing NEDD8 levels may even promote tumor growth. NEDDylated PTEN has been detected in breast cancer cells lines and NEDD8 conjugation to PTEN allows for nuclear localization and promotes tumor growth [107]. A recent study demonstrated that NEDD8 can directly bind to a variety of destabilized, non-canonical, substrates present in cancers characterized by microsatellite instability [108]. NEDD8-conjugation promoted the degradation of destabilized substrates and treatment with MLN4924 led to the accumulation of misfolded proteins within the cell. Interestingly, combining MLN4924 treatment with immune checkpoint-inhibitor treatments led to better outcomes in murine models of microsatellite instable tumors [108].

**Figure 2:**
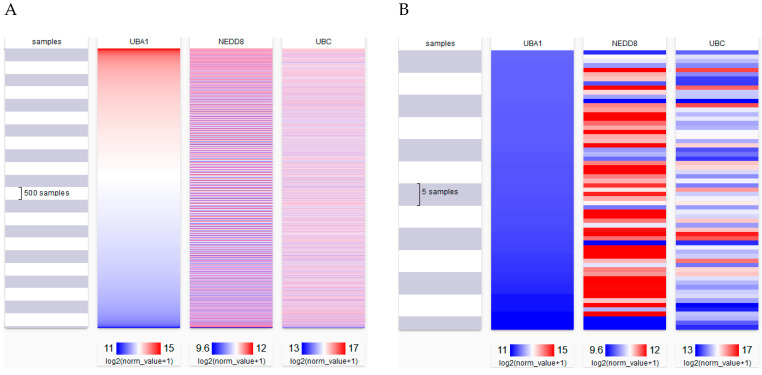
The TCGA Pan-Cancer was used to analyze 11,072 tumor samples for expression levels of UBA1, NEDD8, and UBC. Samples were ranked based upon UBA1 expression and visualized using Xena. (**A**) Visualization of total samples analyzed. (**B**) Visualization of 63 samples with the lowest level of UBA1 expression.

To determine if the conditions for atypical NEDDylation existed on a more global scale, we analyzed The Cancer Genome Atlas (TCGA) data sets to compare transcript levels of NEDD8 as well as UBA1 and Ubiquitin C (UBC) in ~12,000 samples for which data were available. We rationalized that if levels of UBA1 and ubiquitin decreased while levels of NEDD8 increased, then a favorable environment would exist for atypical NEDDylation. The data were visualized using the Xena Platform [109]. As shown in Figure 2, there does not appear to be a strong correlation between expression of the three genes across the entire dataset; however, there are certainly some samples in the database showing relatively low levels of UBA1 and UBC while NEDD8 levels are much higher (Figure 2B), which resembles the conditions under which atypical NEDDylation of non-Cullin substrates have been identified in vitro. To simplify the analysis and look for statistical significance, we compared levels of UBA1 expression with NEDD8 to determine if there was a correlation both in the pan-cancer dataset as well as specific tumor types. When samples from all tumor types were included, there was a slight, but significant, positive correlation between UBA1 and NEDD8 expression (R = 0.134), which would suggest that in many cancers as NEDD8 levels increase there is also an increase in UBA1 (Table 1). However, in certain cancer types, there were statistically significant negative correlations between UBA1 and NEDD8 expression, including colon and rectal cancers (R = −0.1414) and lung adenocarcinoma (R = −0.1983). Glioblastoma had the greatest negative correlation between UBA1 and NEDD8 (R = −0.5184), consistent with reports showing up-regulation of NEDD8 in glioblastoma [110]. In these particular tumor types, it would appear that increased levels of NEDD8 and/or decreased levels of UBA1 are present and could in fact be representative of the conditions used to isolate NEDDylated proteins in vitro. Therefore, we should not rush to dismiss the non-canonical substrates of NEDD8 as artifacts when in fact they may be quite important in clinically relevant settings.

## 9. NEDD8 Chains Bind to Non-Canonical Substrates

Recent reports highlight the importance of considering not only mono-NEDDylation but the importance of NEDD8 chains attached to non-Cullin substrates and their biological roles in disease. Rnf111 was shown to add multiple NEDD8 residues to the C-terminus of histone H4 at sites of DNA damage [111]. Bailly et al. found that HSP70 can bind to NEDD8 chains and following DNA damage elevated levels of the de-NEDDylase NEDP1 cleaves the NEDD8 chain from HSP70, resulting in its mono-NEDDylation [112]. Mono-NEDDylated HSP70 can then promote the oligomerization of apoptotic protease activating factor 1, resulting in the induction of apoptosis. Interestingly, the authors found that NEDP1 was down-regulated in a moue model of hepatocellular carcinoma suggesting that in tumors, the increased levels of NEDD8 chains on HSP70 prevents tumor cell apoptosis. NEDP1 was also shown to remove a trimeric NEDD8 chain from the pro-apoptotic protein poly(ADP-ribose) polymerase 1 (PARP-1), leading to the activation of PARP-1 and induction of apoptosis [113]. Additionally, NEDP1 was down-regulated following H_2_O_2_-induced oxidative stress, leading Keuss et al. to conclude that oxidative stressleaves PARP-1 in its inactive state and prevents apoptosis allowing the cell to recover from the stressor without dying [113]. These reports point to the importance of discovering not only if non-canonical substrates are NEDDylated, but also the importance of the types of NEDD8 chains which may bind to such substrates.

## 10. Conclusions and Future Perspectives

To summarize, NEDD8 conjugation to non-canonical substrates has been detected in numerous studies, but the consequences of NEDDylation are not always consistent. This contrasts with the canonical NEDD8 substrates, the CRLs, where NEDD8 conjugation allows the CRL to ubiquitinate the substrates for which it is specific. The results of NEDDylation of non-CRLs can be quite varied, and could result in degradation of the substrate, increased stabilization of the substrate, and sequestration of the substrate in particular regions of the cell including both transient structures such as stress granules, or more stable structures such as the nucleolus. NEDDylation of non-CRL substrates is likely to have meaningful, physiological effects in disease when NEDD8 is dysregulated, which has been observed in several cancer types.

Biological systems are often complex and our desire to simplify them can often lead us astray. Trying to rationalize general rules on how ubiquitin and ubiquitin-like protein modifications alter a proteins function can often feel like we are wrapping ourselves tighter and tighter into a cocoon from which escape seem impossible, fulfilling Webster’s warning of tangling ourselves in our work like the silkworm. Indeed, to extend the metaphor to a biological setting, the substrates of NEDD8 were recently identified in the silkworm *Bombyx mori* and of the 58 proteins identified, none were Cullins [114]. Therefore, it is important to continue to describe when and how modifications of ubiquitin and UBLs exist and how the post-translational modification with ubiquitin and UBLs alters a substrates function. Attention should be paid to any potential stressors placed upon cells, as stress may impact NEDDylation of non-canonical substrates in different ways. The field should also attempt to follow the fate of the substrate to determine its stability, activity, and localization following the addition of NEDD8. As these studies continue, we may, perhaps, be able to find the common patterns and untangle our knowledge.

## Figures and Tables

**Table 1:** Comparison of UBA1 and NEDD8 expression in different TCGA samples sets. The regression analysis and statistical significance are noted.

TCGA Set	R Value	*p* Value
Pan-Cancer	0.1034	1.172 × 10^−27^
Colon and Rectal	−0.1414	0.003146
Colon	−0.1422	0.009796
Glioblastoma	−0.5184	3.319 × 10^−13^
Head and Neck	−0.1160	0.005715
Lung Adenocarcinoma	−0.1983	0.000001616
Lung Cancer	−0.1608	5.586 × 10^−8^
